# Assessments of Antibacterial Effects of Aqueous-Ethanolic Extracts of* Sida rhombifolia*'s Aerial Part

**DOI:** 10.1155/2018/8429809

**Published:** 2018-12-18

**Authors:** Demeke Debalke, Mastewal Birhan, Amebaye Kinubeh, Muluken Yayeh

**Affiliations:** Department of veterinary Paraclinical studies, College of Veterinary Medicine and Animal Sciences, University of Gondar, Gondar, Ethiopia

## Abstract

Infectious diseases are the critical problems of the world as a result of the emergence of different antimicrobial resistant microorganisms due to several reasons like misuses and repeating uses of antibiotics. Because of this, searching of new treatment method is important from natural substances to against those infectious diseases in both human and animals' aspects. Among those plants,* Sida rhombifolia* has various roles against those infectious diseases through its different phytochemical components. The objectives of this study were assessing the antibacterial activity of the aqueous-methanol extract of the plant's aerial part and knowing the phytochemical constituents of the plant. Preliminary phytochemical screening revealed that the extract of* S. rhombifolia*'s aerial part possesses flavonoids, alkaloids, polyphenols, and quinines. In addition to this, the antibacterial activity of the plant extract was evaluated on five pathogenic bacteria species using agar well diffusion method at different concentrations of plant extracts. Minimum inhibition concentration and minimum bactericidal concentration determinations were done by tetrazolium chloride microtiter dilution assay. The inhibition zone of mean diameters ranging from 0.00 to 7.67mm against all test bacteria was significantly (p<0.05) much less than that of the positive control Chloroamphinicole (30*μ*g/disc) with the range of 14.33mm-15mm of inhibition zone of diameters. The inhibition zones of the tested bacteria at the concentration of 62.5mg/ml were much less than the higher concentration (500mg/ml) and significantly different (p<0.05), whereas the MIC value ranges from 4.62 to 97.22mg/ml and the MBC value ranges from 4.62 to 125.00mg/ml. Even if the plant extract showed antibacterial activity, it was lower than that of other solvent extraction methods; so other solvent extraction methods and fractionates must be conducted to investigate the antibacterial activities of the plant extract on different bacterial strains and species that cause different diseases.

## 1. Introduction

In developing African countries like Ethiopia, livestock production remains essential and represents a major asset among resource-poor smallholder farmers by giving milk, meat, skin, manure, and traction. However, the economic benefits of livestock populations remain minimal due to prevailing livestock diseases, which are among the principal obstacles for livestock performance and cause of high economic losses of the resource poor farmers [1]. This condition has been aggravated by the inadequate provision of modern medicines, which in turn is caused by lack of sufficient money to import those medicines by the government and the emergence of resistant strains of pathogens to the antibiotics [2].

In Ethiopia, currently livestock population estimate stands at 59.5 million cattle, 30.70 million sheep, 30.20 million goats, 1.21 million camels, 8.44 million donkeys, 2.16 million horses, 0.41 million mules, and 56.53 million poultry. According to the survey result, about 87.85 million chickens, 11.98 million sheep, 12.29 million goats, and 10.98 million cattle were born during the reference period in the rural sedentary areas of the country [3].

Use of natural products for curing wide variety of human and domestic animal diseases has a long history that goes to human civilization [4]. Medicinal plants are the backbone of traditional medicines and variety of bioactive substances present in these plants are widely used against various diseases [5]. About 25% of prescribed drugs in the world are of plant origin [6]. From the ancient period, different parts of medicinal plants have been used for ailments caused by microorganisms. There is a wide range of medicinal plant parts possessing a variety of pharmacological activities, such as flowers, leaves, barks, stems, fruits, and roots extracts which are used as powerful raw drug. Recently, there is a widespread interest of plants derived drugs that reflect its recognition of the validity of many traditional claims regarding the value of natural products in healthcare [7].

Over the last few decades, a great interest has developed in searching for antimicrobial drugs from natural products due to the belief that drugs derived from plants are safe and dependable compared with synthetic drugs that may have adverse effects on host besides their high cost [8]. Currently, many bacterial pathogens are becoming resistant to the existing antibiotics due to their misuse or repeated use of antibiotics in the treatment of infectious diseases; because of these, scientists advance in their research findings on the bacterial targets to attack the evolved bacteria and attentions towards to the popular plant extracts and biologically active compounds isolated from the plant [9].

Ethiopia's climatic condition is favorable for growing of many medicinal plants, so Ethiopian biodiversity has excellent research and development opportunity for medicinal plants [10]. In Ethiopia, plant remedies are still the most important and sometimes the only sources of therapeutics for nearly 80% of humans and more than 90% in livestock population. Despite their vital role in catering for the health of human and livestock population, large part of the knowledge of ethnomedicinal plants is on the verge of irreversible loss and is declining to deterioration due to oral passage of herbal heritage from generation to generation verbally rather than in writings [11].

Traditional knowledge regarding medicinal plants and their use by indigenous cultures is useful not only for conservation of cultural traditions and biodiversity but also for community healthcare and drug development in the present and future. Plant and plant extracts have formed important position in modern medicine, due to their chemical and medicinal contents found in the natural form. The secondary metabolites represent a large reservoir of structural moieties which work together exhibiting a wide range of biological activities [12]. The crude extracts of plant parts and phytochemicals are known to contain some biological properties and can be of great significance in the therapeutic treatments [13].

Malvaceae is a cosmopolitan family of herbs, shrubs, and trees. Modern research carried out on the Malvaceae plants showed that most of the plants belonging to this family are medicinally important as they contain biologically active compounds [14].* Sida rhombifolia* grows in tropical and warm regions and is distributed throughout the tropics and is known for its wide range of medicinal uses [4]. The dialect name of S.* rhombifolia* is “Gorjejit” in Amharic and “Aratha” in Jimma [15] and different names are given in different localities of English speakers, such as Queensland* hemp*,* sida hemp*,* Cuba jute*,* arrow leaf sida*, and* broom jute sida*, and in French speakers, Chanvre du Queensland and herbedure [16].

*Sida rhombifolia *has considerable reputation for its medicinal value in traditional medicine. The plant is much used for poulticing ulcers, boils, swellings, broken bones, cuts, herpes, and styles and for a skin application in chicken pox. The roots and stems are useful in fever, heart disease, piles, and all kinds of inflammation. Stem is also employed as demulescent and emollient [17].* Sida rhombifolia* possesses pharmacological properties such as antimalarial properties, antibacterial, antiviral activities, and hepatoprotective, anti-inflammatory, and analgesic properties, and phytochemical analysis of the aerial parts of it showed the presence of flavonoids, tannins, sterols, triterpenes, and volatile oils [18].* Sida rhombifolia* herbs in “Ari” local area of Ethiopia used for treatment of calves' constipation by leaves with succulent stem are chopped, mixed with water, and administered orally [19]. Because of these functions and others, the studies of the plants characteristics were needed in the past and are needed in the future. Then the objectives of this study are as follows:

- To evaluate and assess the antibacterial activity of* Sida rhombifolia* aerial parts of aqueous-methanol extract
- To assess phytochemicals of aqueous-ethanolic extract of* Sida rhombifolia* aerial part

## 2. Materials and Methods

### 2.1. Collection and Identification of the Plant Material

Fresh whole parts of* Sida rhombifolia* used for this experiment were collected from natural habitats in Addis Zeeman which is found in Gondar, in October 2017. The identification of the plant sample was authenticated at the University of Gondar, College of Computational and Natural Sciences, Department of Biology.

### 2.2. Preparation of the Plant for Extraction

The collected plant material was dried at room temperature without exposing it to direct sun light in shaded area so as to bring down the initial large moisture content for about 3 weeks. The aerial parts of the plant material were cut by scalpel blade with the help of hand and the dried material was then ground using a mechanical grinder to form fine powder material of the plant. About 280gm powder of aerial plant material was gained after the plant material was ground. Then the powdered material was extracted with methanol and water combination (80%: 20%) within two days' interval for three times and shaken by hand for mixing purpose. The extracted result solution had been filtered using clean double gauze into clean flask and stored in +4°C. Then the filtrated solution was evaporated by rotary evaporator with 70°C temperature and stored in +4°C with sterilized flask until use for experiments [20].

### 2.3. Phytochemical Screening

The aqueous-methanol extract of* Sida rhombifolia* aerial part was used for phytochemical screening. To identify the chemical constituents of plant extract, standard procedures were followed. The plant was screened for alkaloids, saponins, flavonoids, cardiac glycosides, polyphenols, quinines, and terpenoides as the following procedures proceed.

#### 2.3.1. Test for Saponins

To 0.5ml of the extract of plant materials in a test tube, 5 ml of distilled water was added and the mixture was vigorously shaken. Formation of froth persistent for 30 minutes confirms the presence of saponins [21].

#### 2.3.2. Test for Flavonoids

About 10 ml of ethyl acetate was added to 0.25ml of the extract and heated on a water bath for 3 minutes properly. The mixture of the plant extract and ethyl acetate was cooled and filtered. Then, about 4 ml of the filtrate was taken and shaken with 1 ml of dilute ammonia solution. The layers were allowed to separate and the yellow color in the ammonial layer indicated the presence of flavonoids [22].

#### 2.3.3. Test for Cardiac Glycosides (Keller-Killiani Test)

To 0.25gm of the extract diluted to 5 ml in water, 2 ml of glacial acetic acid containing one drop of ferric chloride solution was added. This was underlied with 1 ml of concentrated sulfuric acid. A brown ring at the interface indicated the presence of a deoxysugar characteristic of cardenolides. A violet ring may appear below the brown ring, while in the acetic acid layer, a greenish ring may form just above the brown ring and gradually spread throughout this layer [22].

#### 2.3.4. Test for Polyphenols

To 5 ml of the aqueous solution of the extract, 1 ml of FeCl_3_ (1%) and 1 ml of K_3_ (Fe (CN)_6_) (1%) were added. The appearance of fresh reddish blue color indicated the presence of polyphenols (Farhan* et al*., 2012).

#### 2.3.5. Test for Terpenoides

To 0.25gm of the extract of* Sida rhombifolia*, 2 ml of chloroform was added. Then, 3 ml concentrated sulfuric acid was carefully added to form a layer. A reddish brown coloration indicated the presence of terpenoides in the plant extract [22].

#### 2.3.6. Test for Alkaloids

About 0.25gm of the extract was stirred with 5 ml of 1%HCl on water bath. One milliliter of the filtrate was treated with a few drops of Mayer's reagent and another 1 ml was similarly treated with Dragendorff's reagent. Turbidity or precipitation with both reagents was taken as preliminary evidence for the presence of alkaloids [22].

#### 2.3.7. Test for Quinines

To test the quinine phytochemical presence, in a test tube, 1 ml of extract and 1 ml of concentrated sulfuric acid (H_2_SO_4_) were added. Formation of red color indicates the presence of quinine [23].

### 2.4. Evaluation of Antibacterial Activities

#### 2.4.1. Inoculum Preparation and Preparation of Test Solutions

The tested microorganisms were separately cultured on sterilized Muller-Hinton Agar (MHA) at 37°C for 24 hours by using streak plate method. Then, well-isolated overnight-cultured colonies of the same morphological type were selected from the cultured media. Each colony was touched with a flamed wire-loop and the growth was transferred into a sterilized test tube containing 5 ml steriled normal saline solution. The test tubes that contain the bacterial suspension were vortexed to be mixed well uniformly. Then, the bacterial suspension was adjusted with 0.5 McFarland turbidity standards. The adjustment and comparison of turbidity of Inoculum tubes were performed by visually observing them with naked eye against a 0.5 McFarland turbidity equivalence standard with white background and contrasting blue lines in the presence of adequate light. The adjusted bacterial suspensions should be used as inocula within 15 minutes; otherwise, they are not used for testing purpose.

#### 2.4.2. Tested Microorganisms

A total of 5 bacterial microorganisms were used in this experiment for zone of inhibition and MIC and MBC assays. The bacteria were purchased from National Veterinary Institute (NVI), Bishoftu, Ethiopia, and gained from National Animal Health Diagnostic and Investigation Center (NAHDIC), Sebeta, Ethiopia. The bacterial strains under the study include* Escherichia coli* (*E. coli*) (ATCC-27853),* Salmonella typhi* (*S. typhi*) (ATCC -13062),* Staphylococcus aureus* (*S. aureus*) (ATCC- 2529),* K. pneumonia*, and* Citrobacter*.

#### 2.4.3. Determination of Zone of Inhibition (Zone of Inhibition Test)

For the determination of zone of inhibition, Chloroamphinicole (30*μ*g/disc) was taken as standard antibiotic for comparison of the results. The antibacterial activities of the crude aqueous-methanol extract of the aerial part of* S. rhombifolia *were tested against one Gram-positive and four Gram-negative bacterial microorganisms. The zone of inhibition test was done by agar well diffusion method described by [24].

Pathogenic test bacteria were streaked to MHA plate and incubated at 37°C for 24 hrs. Before this, suspensions of the bacterial isolates were made in sterile normal saline and adjusted to the 0.5* McFarland's* standard solution. Small volumes of bacterial suspensions were swabbed to each MHA plate and then evenly seeded and streaked by means of sterile cotton swab on the agar plate surface. This procedure was repeated by streaking two or more times, rotating the plate approximately 60° each time to ensure an even distribution of inoculums, and, finally, the rim of the agar was swabbed.

Then the agar wells were prepared by using a sterilized cork borer with 6 mm diameter [24]. By using a micropipette, four different concentrations of the plant extract solutions (500mg/ml, 250 mg/ml, 125 mg/ml, and 62.5mg/ml of plant extracts were mixed with 5% of DMSO) and 5%DMSO were carefully added to the respective wells in the plate media and performed in triplicate. The antibiotic disc (Chloroamphinicole 30 *μ*g/disc) was dispensed with a dispensing apparatus (sterile pair of forceps) onto the surface of the inoculated agar plate and pressed down to ensure complete contact with the agar surface.

The plant extracts and antibiotic disc were allowed to diffuse for about one hour before incubation and then the plates were incubated in an upright position at 37°C for 24 hrs. After overnight incubation, the diameters of inhibition zones were measured in mm using Caliber and the results were recorded separately. Chloroamphinicole (30*μ*g/ml) served as positive control separately, while 5% DMSO was used as negative control.

#### 2.4.4. Determination of the Minimum Inhibition Concentration (MIC) by 96-Well Microtiter Plate Using Tetrazolium Chloride

The minimum inhibitory concentration (MIC) for plant extract was evaluated according to method described by [25] with minor modification employing 96-well micro plates. For each plate 50 *μ*l of MHB was placed to each well followed by 100 *μ*l of plant extract (which contains 500 mg/ml of plant extract) added to the first column of the microplates. This made each well of the first column have a total volume of 200 *μ*l. Starting from the first column serial dilution was conducted up to 10th column with double folding; the final volume (50*μ*l) of the plant extract and the broth were drawn from the 10th column. One milliliter of MHB was mixed with 100 *μ*l of bacterial suspension from which 50 *μ*l was filled into the wells up to 10th column aseptically. Subsequently, the plates were incubated for 24 hrs at 37°C incubator. The minimum inhibitory concentration (MIC) was determined by adding 30 *μ*l (2 mg/ml) of 0.02% p-iodonitrotetrazolium chloride (INT) and incubated at 37°C for 30 minutes. INT was used as an indicator for bacteria growth; bacteria metabolize it and changed into pink color. The wells that had no change in color after the addition of INT indicated no growth of the microorganisms and they were taken as MIC values.

#### 2.4.5. Determination of Minimum Bactericidal Concentration (MBC)

The MBC is defined as the lowest concentration where no bacterial growth is observed. This was determined by aseptically subculturing the contents of wells from the MIC results for individual bacterium to antimicrobial-free agar as described in [26].

In this technique, the contents of wells containing a concentration of test material of the MIC value from each four wells, in the MIC determination microdilution test, were dropped using a micropipette about 3 *μ*l on colony counting agar media aseptically and incubated at 37°C for 24 hrs and checked whether there was presence of growth or not. The lowest concentration of the extract that showed no bacterial growth after incubation was observed for each of the four well contents and noted as the MBC. The test was done in triplicate.

### 2.5. Data Analysis

The experimental data are expressed as mean ± standard error of the mean (SEM). Data were analyzed using the Statistical Package for Social Sciences (SPSS) (version 20.0) software. The statistical difference of the mean zone of inhibition of the extract for individual bacterium was carried out by employing one-way analysis of variance (ANOVA) followed by Tukey's post hoc multiple comparison test at a significance level of p<0.05. The MIC and MBC are analyzed using one-way analysis of variance (ANOVA) using SPSS software.

## 3. Results

### 3.1. Phytochemical Screening

The phytochemical screening of the extract of* S*.* rhombifolia*'s aqueous-methanol extract indicated the presence of terpenoides, alkaloids, quinine, polyphenols, and flavonoids. However, saponins and cardiac glycosides were absent in the plant extract as shown in Table 1.

### 3.2. Antibacterial Activity Test

The zones inhibition of mean diameter of the plant extract against the tested bacteria is tabulated in Table 2.

Among the tested bacteria,* K*.* pneumonia* (7.67mm) was moderately susceptible compared to the other tested bacteria within the concentration of 500 mg/ml of plant extract of aqueous ethanol of* Sida rhombifolia*'s aerial part in this study. As depicted in Table 2, the moderate susceptible bacterium at 500 mg/ml was* K*.* pneumonia*(7.67mm) followed by* Citrobacter*,* S*.* typhi*, and* S*.* aureus* with a mean of zone of inhibition diameters (7.1mm, 7.1mm, and 6 mm), respectively. From the tested bacteria,* E. coli* is slightly resistant to the plant extract at the concentration of 500 mg/ml with 5 mm mean of inhibition zone diameter; even in the lower plant extract, concentrations showed lower inhibition zone of diameter. At the concentration of 500 mg/ml of the plant extract, the antibacterial inhibition activities were nearly similar for the tested bacteria.

Based on the mean value zone of inhibition, the plant extracts' antibacterial activity ability was depending on the concentrations of the plant extracts used. At the concentration of 62.5mg/ml of plant extract,* S. aureus* had no inhibition zone, whereas other bacteria show little mean zone of inhibition diameter in the same concentration (62.5mg/ml).

The zones of inhibition of the extract at 250 mg/ml were significantly different when compared to those of 62.5mg/ml and positive control (p<0.05) for all tested bacteria except that of* K*.* pneumonia *(p>0.05). The zones of inhibition of the extract at 250 mg/ml were significantly different when compared to that of 500 mg/ml for* Citrobacter*,* S*.* aureus*, and* S*.* typhi* (p<0.05). However, the plant extracts' zones of inhibition diameter at the concentration of 125 mg/ml were not significantly different when compared to 62.5mg/ml of plant concentration for bacteria* E*.* coli*,* S*.* aureus*,* S*.* typhi*,* Citrobacter*, and* K*.* pneumonia* (p>0.05).

The zone of inhibition of the plant extract at the concentration of 62.5mg/ml was significantly different when compared to 500 mg/ml of the plant extract for the tested bacteria* S*.* aureus*,* E*.* coli*,* K*.* pneumonia*,* S*.* typhi*, and* Citrobacter* (p<0.05) and the zone of inhibition of the plant extract at 500 mg/ml was significantly different from that of its activity at 125 mg/ml concentrations against all tested bacteria (p<0.05). All the tested bacteria in all concentrations of plant extract were significantly different from that of positive control (Chloroamphinicole, 30 *μ*g/disc) (p<0.05). Generally, zones of inhibition mean of the antibacterial activity of the plant extract was depending on its concentrations.

### 3.3. Minimum Inhibition Concentration (MIC) and Minimum Bactericidal Concentration (MBC)

The MIC figure of the plant extract was in agreement with its zones of inhibition of antibacterial activity in the case of* K. pneumonia* but for the other bacteria slightly agreed with the preliminary zone of inhibition mean diameter which means having higher inhibition and lower concentration.

The MIC and MBC of the tested bacteria were equal in the case of* S*.* aureus*,* K*.* pneumonia*, and* S*.* typhi* with the mean concentrations of 32.40mg/ml, 4.62mg/ml, and 32.40mg/ml, respectively, for both assays. The MIC and MBC values for the bacteria* E. coli* and* Citrobacter* were with the figures of 97.22mg/ml (125mg/ml) and 23.14mg/ml (13.88mg/ml), respectively, for individual values.

As indicated in Table 3, the plant extract of aqueous methanol had the maximum MIC of 97.22mg/ml (against* E. coli*) and the minimum MIC value was 4.62mg/ml against* K*.* pneumonia*. Moreover, unlike the slight differences between the figures of zone of inhibition of the extract for S.* aureus* and* S. typhi*, their MIC values were equal. However, in the case of* S*.* typhi and Citrobacter* having similar zones of inhibition figures, their MIC values were different (32.40mg/ml> 13.88mg/ml). The MIC values order for all tested bacteria starting from the higher to the lower were 97.22mg/ml, 32.40mg/ml, 32.40mg/ml, 13.88mg/ml, and 4.62mg/ml with the respective bacteria (*E. coli, S. aureus, S. typhi, Citrobacter, and K. pneumonia*), respectively, whereas the MBC value also had the same order in the figures of 125 mg/ml, 32.40mg/ml, 32.40mg/ml, 23.14mg/ml, and 4.62mg/ml, respectively, for the tested bacteria (*E. coli, S. aureus, S. typhi, Citrobacter, and K. pneumonia*).

## 4. Discussions

Medicinal plants are used for treatments of infectious diseases which are caused by different pathogenic microorganisms and are becoming worldwide threats. Among these microorganisms, bacteria are the most and very hazardous microorganisms in present time. As the result of those characteristics, treatment options should be changed to tackle these difficulties. Infectious diseases are one of the major problems in developing as well as developed countries. Traditional medicinal plants are widely used to treat the microbial diseases due to their rich source of antimicrobial activity and less cost [27]. The present study permitted the evaluation of some biological properties of* S. rhombifolia*, including the antibacterial activity of some selected bacterial species.

Groups of phytochemical compounds commonly associated with combating microbial resistance and having antimicrobial activity in medicinal plants are flavonoids, alkaloids, tannins, triterpenoids, essential oils, saponins, glycosides, and phenols [28]. Even though at this point in time it is difficult to judge the mechanism of actions of the bioactivity of the extract of the study plant of the* Sida rhombifolia*'s aerial part, it is possible to say that the plant has antibacterial activity based on the chemical detection methods in the phytochemical screening of aqueous-ethanol extract.

Preliminary phytochemical analysis of the ethanol extract of fruits of* S. rhombifolia *revealed the presence of tannins, phenolics, and flavonoids [29] and the aqueous-methanol extract of this plant indicated the presence of tannins, polyphenols, alkaloids, glycosides, flavonoids, and saponins [30]; the antibacterial activity of the plant extract in this study can be attributed to the presence of various bioactive components such as alkaloids, flavonoids, terpenoides, polyphenols, and quinine found in this aqueous-methanol extract based on the preliminary test analysis. So, these and other phytochemical constituents of the plant extract may help its antibacterial activity.

The age of the plant and the plant part used for extraction are important parameters, which can affect the ethnopharmacological activity of the extract. In case of alkaloids, older plants have much less alkaloids compared to the younger plants [31]. Phytochemical constitutes of the plant extracts depend on the age of the plants that are used in the study. The presence of flavonoids that are exerted their multiple biological properties includes antimicrobial, cytotoxic, act as powerful antioxidants activity and anti-inflammatory [32]. In the other cases, polyphenolics have been reported to possess antibacterial activity [29]. In the present study's plant extract, also there were flavonoids which contribute to the antibacterial activity of the plant.

Currently, many of the pathogenic bacteria have become multidrug resistant to commonly used antibiotics causing different diseases. Because of this, searching of new drugs is important in the recent times and* Sida rhombifolia* has been proven as a potential source of bioactive molecules [17]. The diameter inhibition zones of the aqueous-methanol extract (20%:80%) in this study range from 00.00 to 7.67mm. However, in the previous study, there was a report of much greater zone of inhibition of diameters than that in this study about 8.7-23.6mm. These results were not consistent with previous reports describing antibacterial activities of* Sida rhombifolia*'s aerial plant extract of aqueous ethanol and methanol [30]. Based on this report, the present study's plant extract has no agreement. These greater differences of zone of inhibition maybe come from the differences in antibacterial activity in that geographical area of plant collection, bacterial strains used, and the parts of the plant used for extraction purpose (Berhanu, 2014). The other work suggested that the small proportion of water in the mixture solvent (1v:4v) may increase the quality or the quantity of the phytochemical bioactive components of the plant extract [33]. But in the present study's plant, it does not coincide. The other reason may be also that most of the bacterial species used in this study were Gram-negative except* S*.* aureus*. Because the antibacterial activity increased due to the presence of the bioactive plant molecules, for Gram-negative bacteria, the plant extracts individually showed less antibacterial activities than those of Gram-positive bacteria. This may be due to the presence of an extra outer membrane in Gram-negative bacteria, which consists of lipopolysaccharide and makes them impermeable to lipophilics, whereas, for the Gram-positive bacteria, they have only an outer peptidoglycan layer that is not an effective permeability barrier [17]. Even if the antibacterial activity of the plant extract was very low in this study, different researches revealed that different plant parts with different solvent extractions have anti-inflammatory and hepatoprotective activities of methanol extracts of aerial part and aqueous extracts of roots and aerial parts [34].

The mean inhibition zones of* S. rhombifolia's aerial part *extract within different concentrations against all tested bacteria were significantly different (p<0.05) and less than the inhibition zones of the positive control Chloroamphinicole (30*μ*g/disc). However,* S. aureus* did not show any zone of inhibition of mean of diameter at the concentration of 62.5mg/ml of the plant extract. Even for the other tested bacteria at the same concentration (62.5mg/ml), little effects were shown. The mean zones of inhibition of the plant extract at the concentration of 500 mg/ml for all the tested bacteria were greater than the lowest concentration of the plant extract (62.5mg/ml) and significantly different (p<0.05).

The antibacterial screening findings in terms of zone of inhibition of the study's plant extract of* Sida rhombifolia *against the respective tested bacteria were inversely proportional to their values of MIC and MBC, which means having higher inhibition zones with lower MIC and MBC figures, with the exception of* Citrobacter.* The MIC range against the tested bacteria was 4.62-125mg/ml for the plant extract in this study but is less than that of a small concentration of 200 *μ*g/ml up to 300 *μ*g/ml of* Sida rhombifolia*, chloroform, and petroleum extracts of the previous study for that of *S*. aureus and* E*.*coli* [29], whereas the other author reported that the MICs of the most sensitive isolates of bacteria to the aqueous-ethanol extracts varied from 49.40 to 78.30*μ*g/ml [30]. This great difference of the growth inhibition of the plant extract for the bacteria may be due to the plant parts used, geographical location of the plant, and bacterial strains used.

All the tested bacteria except* K*.* pneumonia *inhibited their growth with the least dilution, which means high concentrations of the plant extract. However,* K. pneumonia* inhibited its growth with higher serial dilution or with lower concentration of the plant extract even with 4.62mg/ml.

## 5. Conclusion and Recommendations

Medicinal plants are used for treatments of infectious diseases that are caused by different microorganisms. Among these microorganisms, bacteria are the most and very hazardous in present times. The present study revealed that the aqueous-ethanol extract of* Sida rhombifolia's* aerial part has antibacterial activities against the growth of the selected pathogenic bacteria with varying antibacterial spectrum ability even if its inhibition zone of diameters was lower when compared to the other studies on the same plant species with different extraction protocol, bacterial strains, and geographical location of plant collection sites. In the MIC and MBC assays, the plant extract had wide range values of 4.62mg/ml-125mg/ml. The antibacterial activities of the plant might be linked with the presence of alkaloids, terpenoides, flavonoids, and other bioactive phytochemical constitutes in its parts.

Based on the above conclusion, the following recommendations are forwarded:

- The other studies should be done on the other species of bacterial strains, since the extract showed antibacterial activity.
- The plant parts should be extracted with other solvent types to get enough quality and quantity of bioactive molecules.
- The different fractionates of the plant part should be evaluated for the antibacterial activities on different bacteria and other microorganisms.

## Figures and Tables

**Table 1:** Phytochemical screening of *Sida rhombifolia *aqueous-methanol extract of aerial part.

Phytochemical components	Result
Terpenoides	+
Saponins	_
Alkaloids	+
Polyphenols	+
Flavonoids	+
Cardiac glycosides	_
Quinine	+

“+”stands for the presence and “_” stands for the absence of the chemical constitutes.

**Table 2:** Zone of inhibition mean (in mm) of plant extract for tested bacteria.

	Concentration	Tested Bacteria				
		*E. coli*	*Citrobacter*	*S. aureus*	*K. pneumonia*	*S. typhi*
Aqueous-methanol extract	500mg/ml	5.00 ± 0.57^a3d2e3^	7 ± 1.00^a3c1d3e3^	6.00 ± 0.57^a3c2d3^	7.67 ± 0.88^a3d2e2^	7 ± 0.57^a3c2d3e3^
	250mg/ml	3.3.00 ± 0.33^a3e1^	3 ± 1.00^a3e1^	3.33 ± 0.33^a3d1^	5.00 ± 0.577^a3^	4 ± 0.57^a3e1^
	125mg/ml	2.33 ± 0.33^a3^	2.673 ± 0.577^a3^	1.33 ± 0.33^a3^	2 ± 0.57^a3^	2.33 ± 0.33^a3^
	62.5mg/ml	1 ± 0.00^a3^	1.67 ± 0.577^a3^	0.00 ± 0.00^a3^	2.33 ± 0.33^a3^	1.33 ± 0.33^a3^
	Chloram 30*μ*g/disc	15.00 ± 0.57	14 ± 1.00	14.67 ± 0.577	14.33 ± 0.88	14.33 ± 0.33

Values are expressed as Mean ± SEM (n=3); analysis was performed with One-Way ANOVA followed by Tukey test with Post Hoc multiple comparisons; ^a^compared to positive control, ^b^to 500mg/ml, ^c^to 250mg/ml, ^d^to 125mg/ml, and ^e^to 62.5mg/ml; ^1^p<0.0, ^2^p<0.01, and ^3^p<0.001. The negative control has shown no antibacterial activity; Chloram = Chloroamphinicole as positive control; SEM = Standard Error of Mean.

**Table 3:** The MIC and MBC in (mg/ml) of *Sida rhombifolia *aqueous-ethanol aerial part extract against tested bacteria.

Bacteria tested	Extraction **Aqueous-ethanol extract**	
	**MIC**	**MBC**
*E*. *coli*	97.22 ± 27.78	125.00 ± 0.00
*Citrobacter*	13.88 ± 0.00	23.14 ± 9.26
*S*. *aureus*	32.40 ± 9.26	32.40 ± 9.26
*K*. *pneumonia*	4.62 ± 0.00	4.62 ± 0.00
*S*. *typhi*	32.40 ± 9.26	32.40 ± 9.26

MIC: Minimum Inhibitory Concentration and MBC: Minimum Bactericidal Concentration.

**Table 4:** 

Personal protective equipment	Gown, glove, shoes, face mask

Sample preparation materials	Plastic shed, scalpel blade, conical flask, beaker, cotton swab

Laboratory material	Incubator, Petri dish, tree electro balance, refrigerator, autoclave, scalpel blade, tree, aluminum foil, Rota vaporizer machine, pork borer, test tube, petridish, wire loop, Bunsen burner, waste disposal, etc.

Laboratory chemicals and reagents	Alcohol, sulfuric acid, HCl, ethyl acetate, ferric chloride, DMSO, salt (sodium chloride), chloroform, barium chloride, distilled water

Different media and standards	McFarland, media (Muller-Hinton agar), plate count agar, Muller-Hinton broth

## Data Availability

The data used to support the findings of this study are available from the corresponding author upon request.

## Appendix

### A. Material and Equipment

See Table 4.

### B. Procedures for Preparation of Different Reagents and Standards

#### B.1. Procedures to Prepare 0.5 McFarland Standard Solutions

Original* McFarland* standards were made by mixing specified amounts of barium chloride and sulfuric acid together. Mixing the two compounds forms a barium sulfate precipitate, which causes turbidity in the solution. A 0.5 McFarland standard is prepared by mixing 0.05 ml of 1.175% barium chloride dihydrate (BaCl_2_•2H_2_O) with 9.95 ml of 1% sulfuric acid (H_2_SO_4_). The standard can be compared visually to a suspension of bacteria in sterile saline or nutrient broth. If the bacterial suspension is too turbid, it can be diluted with more diluents. If the suspension is not turbid enough, more bacteria can be added.

#### B.2. Preparation of 5% DMSO about 100 ml Solution

- 5ml of dimethyl sulfoxide + 95 ml of distilled water or mostly 5% of DMSO were used.

### C. Procedure of Preparation of Muller-Hinton Agar Media

- 38 gm of the solid medium agar was suspended in one-liter distilled water and mixed thoroughly with the help of hand-shaking several times.
- The mixed medium was heated with frequent agitation and boiled to completely dissolve the components well.
- It was autoclaved at 121°C for about 15 minutes and then cooled to 45°C.
- Then cooled agar was poured into sterile Petri dishes on a level, horizontal surface to give uniform depth of approximately 25 ml of liquid agar for 90 mm plates and waited until it solidified.
- Finally, the prepared media was stored at +4°C or used.
